# Outbreak investigation of extensively drug-resistant *Acinetobacter baumannii* of ST2 in the intensive care unit of a Lebanese hospital

**DOI:** 10.3389/fcimb.2026.1936280

**Published:** 2026-09-24

**Authors:** Tsolaire Sourenian, Marc Finianos, Mohammed Ftouni, Zeinab Najem, Clara Bonaiti, Abdallah Medlej, Jaroslav Hrabak, Costas C. Papagiannitsis, Ibrahim Bitar

**Affiliations:** 1 Department of Microbiology, Faculty of Medicine and University Hospital in Pilsen, Charles University, Pilsen, Czechia; 2 Estonian Genome Center, Institute of Genomics, University of Tartu, Tartu, Estonia; 3 Sheikh Ragheb Harb Hospital, Nabatieh, Lebanon; 4 Pediatric Clinical Research Center Invernizzi, Department of Biomedical and Clinical Sciences, University of Milan, Milan, Italy; 5 Department of Microbiology, University Hospital of Larissa, Larissa, Greece

**Keywords:** *Acinetobacter baumannii*, *bla*_OXA-23_, HAI, outbreak, ST2

## Abstract

**Background:**

The aim of the study is to investigate an XDR *Acinetobacter baumannii* outbreak in the ICU of a tertiary hospital in Lebanon.

**Methods:**

A total of 39 swabs were sampled from the ICU room (n=34; environmental surfaces and n=5; clinical *A. baumannii* swabs from admitted patients). The strains were selected using Mueller Hinton (MH) agars supplemented with 4 µg/mL of meropenem. Species identification and antibiotic susceptibility profiles were determined using MALDI-TOF MS and broth microdilution assays, respectively. Long-read whole-genome sequencing (WGS) was performed on the positive swabs (n=13). Clonal relatedness was assessed by multilocus sequence typing (MLST), and genomes were compared through SNP-based phylogeny.

**Results and discussion:**

Thirteen isolates were XDR *A. baumannii*, of which 8 were from environmental swabs. WGS revealed the presence of chromosomally located *bla*_OXA-23_ gene in all isolates. MLST analysis showed that all strains belong to ST2 international clone. Core-genome SNP analysis using LBN-30 as a reference, resolved the 13 genomes into two outbreak clades and one unrelated genome: Clade A (n=5, 0–1 SNPs from the reference), clade B (n=7, 2248–2387 SNPs from the reference), and LBN-38,the singleton, (2145 SNPs from the reference).

**Conclusion:**

We report a continuous and persistent nosocomial outbreak of XDR *A. baumannii* in the ICU of a Lebanese hospital, despite the implementation of conventional infection control practices. This prolonged circulation of this strain indicates the necessity for extensive systematic screening for both environmental and patient reservoirs, as well as the need for alternative disinfection strategies.

## 1 Background

*Acinetobacter baumannii* is Gram-negative, aerobic coccobacilli that that is ubiquitous in nature and particularly persistent in hospital environments, especially in intensive care units (ICU). It causes a variety of nosocomial infections including septicaemia, pneumonia, endocarditis, meningitis, skin, wound infections, and urinary tract infections (Qi et al., 2016). This species is one of the most notorious opportunistic pathogens associated with high morbidity and mortality rates, and it has been declared by the World Health Organization (WHO) as priority-1 pathogen in 2017 (World Health Organization, 2017).

Multidrug resistance and biofilm formation in *A. baumannii* have made it challenging for clinicians and healthcare providers to treat and control its spread. In *A. baumannii*, resistance to carbapenems has been increasingly reported (Poirel and Nordmann, 2006). This increase may be linked to the production of Ambler class B metallo-β-lactamases (Walsh et al., 2005) as well as carbapenem-hydrolyzing class D β-lactamases (CHDLs) (Naas and Nordmann, 1999). The *bla*_OXA-51_-like genes, which are chromosomally located, encode intrinsic CHDLs of *A. baumannii*. Additionally, three main acquired CHDL-encoding gene clusters, including the *bla*_OXA-23_-, *bla*_OXA-24_-, and *bla*_OXA-58_-like genes, have been identified in *A. baumannii* (Naas and Nordmann, 1999). Although several *bla*_OXA_ genes have been associated with class 1 integrons, the insertion sequence IS*Aba1* has been found upstream of *bla*_OXA-23_-, *bla*_OXA-51_-, and *bla*_OXA-58_-like genes. IS*Aba1*, a member of the IS*4* family of insertion sequences, is involved in the overexpression of the genes located downstream (Mugnier et al., 2009).

Carbapenemase-producing *A. baumannii* isolates exhibit simultaneous resistance to various non-β-lactams antimicrobial agents, limiting the number of efficacious treatment options (Dijkshoorn et al., 2007). Colistin and tigecycline are often the only treatment options for infections caused by multidrug-resistant (MDR) strains. However, recent reports indicate resistance to both agents widespread (Oikonomou et al., 2015; Qureshi et al., 2015). Nevertheless, novel combinations of β-lactam/β-lactamase inhibitor (BL/BLI) have been proposed to treat carbapenem-resistant A. baumannii infections. These are considered a better and safer alternative to colistin (Sargianou et al., 2025). In 2023, sulbactam-durlobactam (SUL-DUR) received FDA approval for hospital-acquired and ventilator-associated bacterial pneumonia. The mechanism of action involves sulbactam retaining intrinsic antibacterial activity while durlobactam inhibits OXA-type carbapenemases (U.S. Food and Drug Administration, 2023). Furthermore, other BL/BLI combinations, such as cefepime-zidebactam, which targets OXA-type enzymes and MBLs, are still under active clinical evaluation (Bassetti et al., 2024). An outbreak of extensively drug-resistant (XDR) *A. baumannii* was detected in the ICU of a tertiary hospital in Lebanon, The outbreak has been enduring for more than four years within the unit despite conventional infection control practices, and incidents have been increasing due to the influx of war casualties. The conflict situation has placed the infection control unit under significant pressure. The isolated section of the ICU containing the three rooms is full, and the hospital is using other sections due to overwhelming numbers and limited capacity. This is leading to minimal isolation procedures due to decreased physical space in the rooms and between patients, decreased screening capabilities due to limited resources, and, finally, the need to transfer patients across multiple facilities and departments, which facilitates dissemination. Finally, according to infection control unit in the hospital, the mortality rate is increasing significantly due to the nature of injuries sustained by war (open wounds), often reported by the hospital in patients developing sepsis and death within 24 hours. Thus, the aim of the study was to trace and identify the particular source of the (XDR) *A. baumannii* outbreak, by combining long-reads sequencing of paired environmental and clinical isolates to establish core-genome SNP phylogeny in order to resolve transmission relationship at single nucleotide resolution.

## 2 Materials and methods

### 2.1 Outbreak description

According to hospital records, a patient was admitted 4 years ago to one of the ICU rooms. After a couple of days, the patient died of sepsis after acquiring an XDR *A. baumannii* in the hospital. After this incident, an increased number of acquired XDR *A. baumannii* associated with the same room (index room) was noticed. Moreover, after a few months, similar cases were observed in two other adjacent ICU rooms. The infection control unit recognized the threat and immediately isolated the 3 rooms from the rest of the ICU. Moreover, surveillance swabs were collected from all inpatients to screen for XDR *A. baumannii.* Patients who were found positive were immediately sent for isolation. After the patients’ discharge or death, the rooms were cleaned and disinfected, and environmental swabs were collected to confirm that they were negative and ready for the next patient. Despite all the measures taken, patients continued to acquire hospital-acquired XDR *A. baumannii* in these 3 ICU rooms. To identify the hidden sources of the outbreak, the index room was selected for screening. The clinical samples in the study were positive swabs from outpatients 4 days preceding the environmental samples, and were included for comparison.

### 2.2 Clinical isolates, identification and susceptibility testing

A total of 39 swabs were sampled from the index ICU room (n=34; environmental surfaces and n=5; clinical *A. baumannii* swabs from admitted patients). The isolates were selected by Mueller Hinton (MH) agars containing 4 µg/mL of meropenem. Species identification of the isolates was performed using matrix-assisted laser desorption ionization-time of flight mass spectrometry (MALDI-TOF MS) through MALDI Biotyper software (Bruker Daltonics, Bremen, Germany). Antimicrobial susceptibility was done using broth microdilution according to EUCAST guidelines (http://www.eucast.org/). Data were interpreted according to the criteria of the European Committee on Antimicrobial Susceptibility Testing (EUCAST v.12) (www.eucast.org).

### 2.3 Whole-genome sequencing and analysis

For the 13 positive swabs, genomic DNA from the *A. baumannii* isolates was extracted using NucleoSpin Microbial DNA kit (Machery-Nagel, Germany). HiFi long-read sequencing was performed using the Vega platform (Pacific Biosciences, California, United States of America); the extracted DNA was subjected to enzymatic shearing and barcoding using the LongPlex multiplexing kit (SEQWELL, Massachusetts, United States of America). Library preparation was then performed using the Microbial multiplexing protocol according to the manufacturer’s recommendation. Using SMRT Link v10.1, the microbial assembly pipeline was used for the assembly and the circularizing of contigs using a minimum seed coverage of 30x.

Assembled sequences were annotated using the NCBI Prokaryotic Genome Annotation Pipeline (PGAP) (Tatusova et al., 2016). Antibiotic-resistant genes, phages, virulence genes, mobile genetic elements, and multilocus sequence types (MLST) were determined through uploading the assembled sequences to ResFinder 4.1 (Bortolaia et al., 2020) and CARD 4.0.1 (Alcock et al., 2023), ISfinder 2.0 (Siguier et al., 2006), and MLST 2.0 (Jolley et al., 2018), as described elsewhere (Sourenian et al., 2024).

### 2.4 Phylogeny and SNP

A total of 2314 *A. baumannii* ST2 genomes were extracted from the NCBI assembly database, including complete and draft genomes. Using Parsnp v1.7.4 (Treangen et al., 2014), a core genome phylogeny of the model General Time Reversible (GTR) based on SNP and recombination, a SNP-based phylogeny was constructed between the sequenced genomes in this study and the 2314 genomes downloaded from the NCBI database as described elsewhere (Sobh et al., 2024). LBN-30 was selected as the mapping reference since it yielded the most contiguous, fully circularized assembly among the rest of the assemblies. Briefly, SNPs identified in local collinear blocks were subsequently used for reconstructing an approximate maximum-likelihood tree using FastTree2 (Price et al., 2010), while including the general time reversible (GTR) model of nucleotide substitution. The Shimodaira–Hasegawa test implemented in FastTree2 was used to assess the support for significant clustering in the observed phylogeny. The interactive tree of life or iTOL 6.4.1 (https://itol.embl.de/) (Letunic and Bork, 2021) was used for the graphic illustration of the trees along with relative annotations.

Moreover, to determine the extent of clonal dissemination in the hospital, SNPs and the pairwise SNP distance between all genomes and SNPs were detected using snippy multicommand (snippy-base application v4.5.0) (Seemann, 2015), which generates a core genome multiple alignment against the common reference (LBN-30). The pipeline detects the variants and generates a single file for each isolate listing the different variations.

### 2.5 Serum resistance assay

The recalcification method, as described previously (Gagaletsios et al., 2025), was used to prepare a pooled human serum from at least 5 different people. Isolates were grown in BHI medium overnight at 37 °C with shaking. The isolates were centrifuged (at 5,000 x g for 15 min), washed twice with PBS, and adjusted to a final concentration of 1*10^7^ CFU/mL in PBS. A total of 1*10^5^ CFU was added to 5 mL of 80% NHS (normal human serum) in PBS and 80% heat-inactivated NHS in PBS. The mixtures were incubated at 37 °C with shaking for 3 h. After incubation, at different time points (0 h and 3 h), the bacterial colony count was determined using 10-fold serial dilutions and conventional plating in Luria Bertani (LB) agar (Miller’s LB AGAR, Condalab). The log values of CFU counts at time 3 h were normalized to CFU counts at time zero were calculated. Moreover, the percentage of CFU recovered at 3h was calculated in percentages compared to the initial one (CFU at 3h/CFU at 0h x 100). Isolates were classified into 3 categories: sensitive (<10% survival), intermediate (10-50% survival), and resistant (>50% survival).

### 2.6 Statistical analysis

Differences in serum survival between clade A (n = 5) and clade B (n = 7) isolates were assessed using the Mann-Whitney U test. The Mann-Whitney U test was used as the data were not assumed to follow a normal distribution, given the small sample sizes. A p-value of <0.05 was considered statistically significant.

## 3 Results and discussion

Thirteen of the thirty-nine swabs (34environmental and 5 clinical) were positive for *A. baumannii* (8 of the 34 environmental and all 5 clinical swabs). Environmental swabs that were positive originated from expected sources (monitors, side rails, air mattress, tray table and toilet chair) and unexpected source (mattress blowing machine (n=1)) (**Supplementary Table S1**). All *A. baumannii* isolates exhibited XDR profiles, showing resistance to ampicillin-sulbactam, piperacillin, piperacillin/tazobactam, ceftazidime, aztreonam, meropenem, gentamicin, amikacin, and ciprofloxacin (**Supplementary Table S2**). Furthermore, 6 isolates were resistant to cotrimoxazole, while only 4 isolates were resistant to colistin.

The genomes of all (n=13) *A. baumannii* isolates were characterized by WGS. Analysis of WGS data with MLST database showed that all strains belong to the ST2 international clone, which corresponds to the international clonal lineage II (Pournaras et al., 2017). Analysis with ResFinder revealed that all isolates carried *bla*_OXA-23_, and *bla*_OXA-51_-like genes. Additionally, all isolates harbored *armA*, *strA*, *strB*, *msr(*E*)*, *mph(*E*)*, and *tet(*B*)* resistance genes. Isolate LBN-38 also carried *aph(*3’*)-Via*, and *sul2* genes, but lacked *tet(*B*)*. The *bla*_OXA-23_ gene was found in variable copy numbers across the isolates:one copy (n=1), two copies (n=5), three copies (n=7).In all isolates, it was carried within the Tn*2006* transposon (Ying et al., 2025), which accounts for both its amplification and insertion at different and multiple chromosomal positions.

SNP-based phylogeny revealed that the isolates recovered from the outbreak clustered into two clades (A and B) (**Figure 1**). Clade A contained 5 isolates, including the one that was used as a reference (LBN-30). Isolates belonging to clade A had 0 to 1 SNPs when compared to the reference genome. Clade B included 7 strains, which differed by 2248 to 2387 SNPs when compared to the reference genome LBN-30 (**Supplementary Tables S3a, S3b**). However, within clade B, isolates differed from each other by 17 to 399 SNPs (**Supplementary Tables S3a, S3b**). Additionally, all isolates of clade B carried 3 *bla*_OXA-23_ copies, while all isolates of clade A harbored two copies of *bla*_OXA-23_. Finally, LBN-38 carried only one *bla*_OXA-23_ copy and differed from clade A isolates by 2145–2146 SNPs and from clade B isolates by 330–503 SNPs. Although closer to clade B, the distance remains above the threshold for nosocomial transmission (<20–25 SNPs for *A. baumannii* as reported by Fitzpatrick et al., 2016 and elsewhere) and grouped with geographically diverse ST2 genomes from the database rather than the outbreak genomes (**Table 1**). The association of *bla*OXA-23 copy number with different clades is of interest, as it highlights the genomic proximity of isolates that may appear phenotypically similar. In this case, the *bla*OXA-23 copy number appears to be associated with the different clones driving this ongoing outbreak.

**Figure 1:**
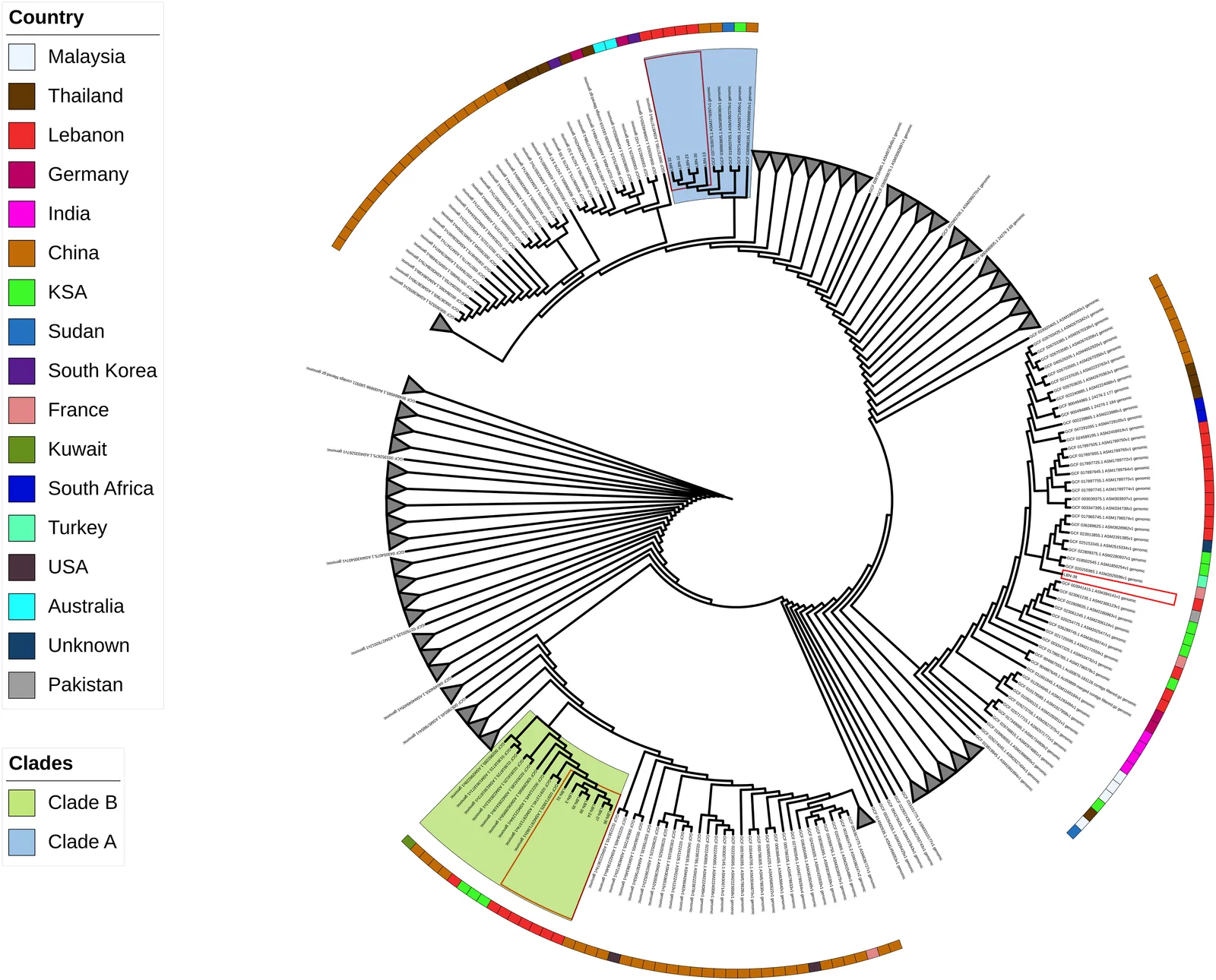
Maximum-likelihood phylogenetic tree constructed from core-genome of all *A. baumannii* ST2 genomes of this study along with 2314 ST2 genomes retrieved from the NCBI database. Genome of LBN-30 was used as a reference. Clade A and B are highlighted in blue and Green respectively. The outermost colored bar to the right corresponds to the isolates’ reporting country. Collapsed nodes (triangle) represent clusters of genomes of high internal similarity.

**Table 1:** Information on isolate sample type in relation with clades and *bla*_OXA-23_ copy number.

Isolate ID	Sample type	Source	ST	Clade	*bla*_OXA-23_	*bla*_OXA-23_ Copy number
LBN-10	Environmental	Monitor	2	A	Yes	2
LBN-13	Environmental	Side rails	2	A	Yes	2
LBN-23	Environmental	Side rails	2	A	Yes	2
LBN-30	Environmental	Air mattress	2	A	Yes	2
LBN-32	Environmental	Mattress blowing machine	2	A	Yes	2
LBN-3	Environmental	Toilet chair + Flash toilet	2	B	Yes	3
LBN-24	Environmental	Tray table + chair	2	B	Yes	3
LBN-31	Environmental	Air mattress	2	B	Yes	3
LBN-35	Patient	Nasal	2	B	Yes	3
LBN-36	Patient	Deep Tracheal Aspirate	2	B	Yes	3
LBN-37	Patient	Nasal	2	B	Yes	3
LBN-39	Patient	Deep Tracheal Aspirate	2	B	Yes	3
LBN-38	Patient	Deep Tracheal Aspirate	2	–	Yes	1

Analysis of WGS data with VFDB, showed that all isolates harbored genes involved in adherence, biofilm formation, immune evasion, iron uptake, serum resistance, and stress adaptation (**Supplementary Table S4**). Of note, isolates of clade A lacked the genes encoding AbaI inducer and its cognate receptor AbaR of Quorum-Sensing system, while clade B isolates missed an open reading frame (ORF) associated with heme utilization. The absence of AbaI/AbaR system may indicate the inability of the bacteria to control a wide variety of processes involved in pathogenicity (Subhadra et al., 2018). Moreover, because virulence genes presence doesn’t always correlate to the phenotypic behavior, we assessed serum survival assay as a phenotypic correlate: the concordance between the absence of AbaI/AbaR quorum sensing system in clade A and its serum sensitive phenotype indicates that the observed genomic difference is actually expressed. The Mann-Whitney statistical analysis was significant (Mann-Whitney U = 0, p-value=0.0025). The isolates belonging to clade A were serum-sensitive, with a median survival rate of 3.1%; those belonging to clade B and the orphane isolate (LBN-38) were intermediate in serum resistance, with a median survival rate of 20.1% and 17% respectively; and one isolate was serum-resistant (52.5%) (**Supplementary Figures S5, S6**).

Several studies have reported similar findings implicating ventilated mattresses and other hidden sources such as feather pillows, and faucets as a persistent reservoirs (Sherertz and Sullivan, 1985; Weernink et al., 1995; Viana et al., 2016; Lv et al., 2019). The department of infection control has been advised to do extensive environmental swabs covering more surface area on the tested objects (multiple swabs from multiple places from the side rails, monitors, etc., beyond the routine practice) to ensure the discovery of any hidden sources. Moreover, failure to properly disinfect may be correlated with: 1- insufficient concentration/low contact time of the disinfectant, 2- inadequate distribution of the disinfectant (objects that may be out of sight where the disinfectant cannot reach), 3- VFDB analysis showed that all isolates harbored genes associated with biofilm formation including biofilm-regulatory and surface attachment determinants; this suggests that the presence of organic load such as dust and secretions (detected while doing the environmental sampling) in which *A. baumannii* may form a niche (extensive pre-cleaning is required). This may be solved by using detergents and microfiber wipes to remove biofilms and organic load from the surfaces, 4- and finally, aerosolized peracetic acid/ozone systems with environmental monitoring may be needed to eradicate bacteria from these possible hidden sources, which is a better option from the one already in use due to the successful persistence of the isolates in environmental settings despite ongoing infection. Moreover, implementation of long reads sequencing has resolved what was dealt with as clonal outbreak into multiple introduction of genetically distant ST2 clones as reported elsewhere (Fitzpatrick et al., 2016; Willems et al., 2016; Mateo-Estrada et al., 2021).

There are two limitations to this study. First, sampling was cross-sectional: environmental and clinical samples at a single point in time were collected during this ongoing four-year outbreak. This design limits our ability to develop an outbreak timeline. As a result, we cannot clearly trace the transmission routes nor the direction of spread across different ICU rooms, nor can we establish the causal relationship between genomic features and clinical outcomes. Second, we lack patients epidemiological variables, including admission dates, length of ICU stay, ward transfer histories, and individual exposure data. Thus, the epidemiological inference is limited, and our genomic findings are considered as supportive but not definitive evidence for the described outbreak dissemination dynamics.

Notwithstanding these limitations, the study identified previously unrecognized hidden reservoirs, demonstrates that the outbreak is prolonged through multiple introduction of genetically distant ST2 clones rather than single clone event as assumed by the infection control unit, and provides a rational basis for targeted control measures to be implemented.

## 4 Conclusion

In conclusion, in the current study, we described the investigation of an outbreak that took place in the ICU of a Lebanese hospital. All isolates belonged to ST2 international clone, which corresponds to the international clonal lineage II, and harbored similar resistomes, including the presence of *bla*_OXA-23_ carbapenemase-encoding gene. Despite all isolates belonging to ST2, SNP-based phylogenetic analysis revealed the presence of two clades, A and B and one clade-orphan genome. Isolates grouped in clade A were of environmental origin, while those in clade B clustered isolates of both environmental and clinical origins. The SNP-based phylogeny suggests that the outbreak originated from multiple introductions of isolates, supported by the presence of two clades and an orphan genome. Nevertheless, this doesn’t refute the possibility of prolonged diversification and microevolutionary events within the isolates, as evidenced by the genomes in clade B.

Examination of *in vitro* virulence combined with VFDB, by testing the survival of bacteria in pooled human serum, showed that isolates of clade A isolates lacked genes encoding AbaI/AbaR quorum-sensing system and were serum sensitive, while clade B isolates exhibited intermediate levels of serum resistance. Bacteria utilize quorum-sensing systems to control a wide variety of processes, which include the synchronized expression of genes involved in colonization, symbiosis, and virulence in host-bacterial infections (Raina et al., 2009). Thus, the lack of *abaI* and *abaR* genes may suggest the inability of the bacteria to control processes involved in the pathogenicity, like serum resistance.

This prolonged circulation of this strain underscores a critical failure in systematic screening for environmental and patient reservoirs and ineffective disinfection strategies. Hence, immediate intervention at the national and hospital levels is demanded to prevent future outbreaks.

## Data Availability

The datasets presented in this study can be found in online repositories. The names of the repository/repositories and accession number(s) can be found in the article/**Supplementary Material**.

## References

[B1] Alcock B. P. Huynh W. Chalil R. Smith K. W. Raphenya A. R. Wlodarski M. A. . (2023). CARD 2023: expanded curation, support for machine learning, and resistome prediction at the Comprehensive Antibiotic Resistance Database. Nucleic Acids Res. 51, D690–D699. doi: 10.1093/nar/gkac920 36263822 PMC9825576

[B2] Bassetti M. Vena A. Larosa B. Giacobbe D. R. (2024). New antibiotics in clinical pipeline for treating infections caused by metallo-β-lactamases producing Gram-negative bacteria. Curr. Opin. Infect. Dis. 37, 582–588. doi: 10.1097/QCO.0000000000001056 39106036 PMC11556884

[B3] Bortolaia V. Kaas R. S. Ruppe E. Roberts M. C. Schwarz S. Cattoir V. . (2020). ResFinder 4.0 for predictions of phenotypes from genotypes. J. Antimicrob. Chemother. 75, 3491–3500. doi: 10.1093/jac/dkaa345 32780112 PMC7662176

[B4] Dijkshoorn L. Nemec A. Seifert H. (2007). An increasing threat in hospitals: multidrug-resistant Acinetobacter baumannii. Nat. Rev. Microbiol. 5, 939–951. doi: 10.1038/nrmicro1789 18007677

[B5] Fitzpatrick M. A. Ozer E. A. Hauser A. R. (2016). Utility of whole-genome sequencing in characterizing Acinetobacter epidemiology and analyzing hospital outbreaks. J. Clin. Microbiol. 54, 593–612. doi: 10.1128/JCM.01818-15 26699703 PMC4767972

[B6] Gagaletsios L. A. Kikidou E. Galbenis C. Bitar I. Papagiannitsis C. C. (2025). Exploring virulence characteristics of clinical Escherichia coli isolates from Greece. Microorganisms 13, 1488. doi: 10.3390/microorganisms13071488 40731998 PMC12300496

[B7] Jolley K. A. Bray J. E. Maiden M. C. J. (2018). Open-access bacterial population genomics: BIGSdb software, the PubMLST.org website and their applications. Wellcome Open Res. 3, 124. doi: 10.12688/wellcomeopenres.14826.1 30345391 PMC6192448

[B8] Letunic I. Bork P. (2021). Interactive Tree Of Life (iTOL) v5: an online tool for phylogenetic tree display and annotation. Nucleic Acids Res. 49, W293–W296. doi: 10.1093/nar/gkab301 33885785 PMC8265157

[B9] Lv Y. Xiang Q. Jin Y. Z. Fang Y. Wu Y. J. Zeng B. . (2019). Faucet aerators as a reservoir for carbapenem-resistant Acinetobacter baumannii: a healthcare-associated infection outbreak in a neurosurgical intensive care unit. Antimicrob. Resist. Infect. Control 8, 205. doi: 10.1186/s13756-019-0635-y 31893039 PMC6938019

[B10] Mateo-Estrada V. Fernández-Vázquez J. L. Moreno-Manjón J. Hernández-González I. L. Rodríguez-Noriega E. Morfín-Otero R. . (2021). Accessory genomic epidemiology of cocirculating Acinetobacter baumannii clones. mSystems 6, e00626-21. doi: 10.1128/mSystems.00626-21 34282943 PMC8407383

[B11] Mugnier P. D. Poirel L. Nordmann P. (2009). Functional analysis of insertion sequence ISAba1, responsible for genomic plasticity of Acinetobacter baumannii. J. Bacteriol. 191, 2414–2418. doi: 10.1128/JB.01258-08 19136598 PMC2655512

[B12] Naas T. Nordmann P. (1999). OXA-type β-lactamases. Curr. Pharm. Des. 5, 865–879. 10539993

[B13] Oikonomou O. Sarrou S. Papagiannitsis C. C. Georgiadou S. Mantzarlis K. Zakynthinos E. . (2015). Rapid dissemination of colistin and carbapenem resistant Acinetobacter baumannii in Central Greece: mechanisms of resistance, molecular identification and epidemiological data. BMC Infect. Dis. 15, 559. doi: 10.1186/s12879-015-1297-x 26653099 PMC4675053

[B14] Poirel L. Nordmann P. (2006). Carbapenem resistance in Acinetobacter baumannii: mechanisms and epidemiology. Clin. Microbiol. Infect. 12, 826–836. doi: 10.1111/j.1469-0691.2006.01456.x 16882287

[B15] Pournaras S. Dafopoulou K. Del Franco M. Zarkotou O. Dimitroulia E. Protonotariou E. . (2017). Predominance of international clone 2 OXA-23-producing Acinetobacter baumannii clinicalin Greece 2015: results of a nationwide study. Int. J. Antimicrob. Agents 49, 749–753. doi: 10.1016/j.ijantimicag.2017.01.028 28427842

[B16] Price M. N. Dehal P. S. Arkin A. P. (2010). FastTree 2—approximately maximum-likelihood trees for large alignments. PloS One 5, e9490. doi: 10.1371/journal.pone.0009490 20224823 PMC2835736

[B17] Qi L. Li H. Zhang C. Liang B. Li J. Wang L. . (2016). Relationship between antibiotic resistance, biofilm formation, and biofilm-specific resistance in Acinetobacter baumannii. Front. Microbiol. 7, 483. doi: 10.3389/fmicb.2016.00483 27148178 PMC4828443

[B18] Qureshi Z. A. Hittle L. E. O’Hara J. A. Rivera J. I. Syed A. Shields R. K. . (2015). Colistin-resistant Acinetobacter baumannii: beyond carbapenem resistance. Clin. Infect. Dis. 60, 1295–1303. doi: 10.1093/cid/civ048 25632010 PMC4462660

[B19] Raina S. De Vizio D. Odell M. Clements M. Vanhulle S. Keshavarz T. (2009). Microbial quorum sensing: a tool or a target for antimicrobial therapy? Biotechnol. Appl. Biochem. 54, 65–84. doi: 10.1042/BA20090072 19594442

[B20] Sargianou M. Stathopoulos P. Vrysis C. Tzvetanova I. D. Falagas M. E. (2025). New β-lactam/β-lactamase inhibitor combination antibiotics. Pathogens 14, 307. doi: 10.3390/pathogens14040307 40333039 PMC12029989

[B21] Seemann T. (2015). Snippy: Rapid Haploid Variant Calling and Core Genome Alignment. Available online at: https://github.com/tseemann/snippy (Accessed May 09, 2025).

[B22] Sherertz R. J. Sullivan M. L. (1985). An outbreak of infections with Acinetobacter calcoaceticus in burn patients: contamination of patients’ mattresses. J. Infect. Dis. 151, 252–258. doi: 10.1093/infdis/151.2.252 3968451

[B23] Siguier P. Pérochon J. Lestrade L. Mahillon J. Chandler M. (2006). ISfinder: the reference centre for bacterial insertion sequences. Nucleic Acids Res. 34, D32–D36. doi: 10.1093/nar/gkj014 16381877 PMC1347377

[B24] Sobh G. Araj G. F. Finianos M. Sourenian T. Hrabak J. Papagiannitsis C. C. . (2024). Molecular characterization of carbapenem and ceftazidime-avibactam-resistant Enterobacterales and horizontal spread of blaNDM-5 gene at a Lebanese medical center. Front. Cell. Infect. Microbiol. 14, 1407246. doi: 10.3389/fcimb.2024.1407246 38962322 PMC11219574

[B25] Sourenian T. Palkovicova J. Papagiannitsis C. C. Dolejska M. Hrabak J. Bitar I. (2024). A novel F type plasmid encoding mcr-10 in a clinical Enterobacter ludwigii strain from a tertiary hospital in the Czech Republic. J. Glob. Antimicrob. Resist. 37, 195–198. doi: 10.1016/j.jgar.2024.03.015 38555080

[B26] Subhadra B. Kim D. H. Woo K. Surendran S. Choi C. H. (2018). Control of biofilm formation in healthcare: recent advances exploiting quorum-sensing interference strategies and multidrug efflux pump inhibitors. Materials (Basel) 11, 1676. doi: 10.3390/ma11091676 30201944 PMC6163278

[B27] Tatusova T. DiCuccio M. Badretdin A. Chetvernin V. Nawrocki E. P. Zaslavsky L. (2016). NCBI prokaryotic genome annotation pipeline. Nucleic Acids Res. 44, 6614–6624. doi: 10.1093/nar/gkw569 27342282 PMC5001611

[B28] Treangen T. J. Ondov B. D. Koren S. Phillippy A. M. (2014). The Harvest suite for rapid core-genome alignment and visualization of thousands of intraspecific microbial genomes. Genome Biol. 15, 524. doi: 10.1186/s13059-014-0524-x 25410596 PMC4262987

[B29] U.S. Food and Drug Administration (2023). FDA Approves New Treatment for Pneumonia Caused by Certain Difficult-to-Treat Bacteria (Silver Spring, MD: U.S. Food and Drug Administration). Available online at: https://www.fda.gov/news-events/press-announcements/fda-approves-new-treatment-pneumonia-caused-certain-difficult-treat-bacteria (Accessed May 09, 2025).

[B30] Viana R. E. H. dos Santos S. G. Oliveira A. C. (2016). Recovery of resistant bacteria from mattresses of patients under contact precautions. Am. J. Infect. Control 44, 465–469. doi: 10.1016/j.ajic.2015.10.027 26739639

[B31] Walsh T. R. Toleman M. A. Poirel L. Nordmann P. (2005). Metallo-beta-lactamases: the quiet before the storm? Clin. Microbiol. Rev. 18, 306–325. doi: 10.1128/CMR.18.2.306-325.2005 15831827 PMC1082798

[B32] Weernink A. Severin W. P. Tjernberg I. Dijkshoorn L. (1995). Pillows, an unexpected source of Acinetobacter. J. Hosp. Infect. 29, 189–193. doi: 10.1016/0195-6701(95)90328-3 7615936

[B33] Willems S. Kampmeier S. Bletz S. Kossow A. Köck R. Kipp F. . (2016). Whole-genome sequencing elucidates epidemiology of nosocomial clusters of Acinetobacter baumannii. J. Clin. Microbiol. 54, 2391–2394. doi: 10.1128/JCM.00721-16 27358465 PMC5005500

[B34] World Health Organization (2017). WHO Publishes List of Bacteria for Which New Antibiotics are Urgently Needed (Geneva: World Health Organization).

[B35] Ying S. Zhang Z. Xiang R. (2025). Metagenomic and whole-genome characterization of carbapenem-resistant Acinetobacter baumannii carrying blaOXA-23 gene within the Tn2006 transposon among ICU patients. J. Glob. Antimicrob. Resist. 44, 180–185. doi: 10.1016/j.jgar.2025.06.009 40555322

